# Pulmonary Embolism in a Young African American Male: A Diagnostic Challenge

**DOI:** 10.7759/cureus.62348

**Published:** 2024-06-14

**Authors:** Moiud Mohyeldin, Vanisa Ezukuse, Ruchi Bhattarai, Rabih Nasr

**Affiliations:** 1 Internal Medicine, BronxCare Health System, Bronx, USA; 2 Department of Medicine, American University of the Caribbean School of Medicine, Cupecoy, SXM; 3 Nephrology, BronxCare Health System, Bronx, USA

**Keywords:** dvt prophylaxis, pulmonary embolism (pe), african american male, in hospital cardiac arrest, thrombo-embolism, massive pulmonary embolism

## Abstract

This case report highlights the diagnostic challenges posed by pulmonary embolism (PE) in a young, otherwise healthy 33-year-old African American male with no apparent risk factors. The patient presented with penile pain, swelling, hematuria, flank pain, and rash, and was admitted for balanoposthitis and acute urinary retention. Despite prophylactic heparin, he suffered two cardiac arrests secondary to PE on the fifth day of hospitalization. Prompt thrombolytic therapy and heparin infusion were initiated, but his course was complicated by anuric acute kidney injury requiring hemodialysis, shock liver, and gastrointestinal bleeding. Imaging revealed a substantial thrombus burden in the pulmonary arteries. Notably, a hypercoagulable workup was negative. The absence of typical risk factors, negative hypercoagulable workup, and occurrence of PE despite prophylaxis underscore the importance of vigilance in recognizing atypical presentations. This case emphasizes the need for a high index of suspicion and comprehensive evaluation to diagnose PE in young patients without clear predisposing factors.

## Introduction

Pulmonary embolism (PE) is a potentially life-threatening condition that occurs when a blood clot becomes lodged in the pulmonary arteries, causing obstruction of blood flow to the lungs [1]. This obstruction can lead to a variety of symptoms, including chest pain, shortness of breath, rapid heart rate, and in severe cases, hemodynamic instability and cardiac arrest [1]. In the United States, the annual incidence of PE is estimated to be 1-2 per 1,000 individuals, with a higher prevalence among older adults and those with predisposing risk factors such as obesity, immobility, surgery, trauma, and inherited thrombophilias [2,3]. However, PE can also occur in young, otherwise healthy individuals without apparent risk factors, making diagnosis challenging [4].

African Americans have been shown to have a higher incidence of venous thromboembolism (VTE), which includes both deep vein thrombosis (DVT) and PE, compared to other racial and ethnic groups in the US [5]. A retrospective study by Zakai et al. found that the age-adjusted incidence of VTE was 30% higher in African Americans than in the White population, with the disparity being most pronounced among young individuals under 40 years of age [6]. The reasons for this increased risk are not fully understood but may involve a complex interplay of genetic, environmental, and socioeconomic factors [7]. Some studies have suggested that African Americans may have higher rates of certain inherited thrombophilias, such as Factor V Leiden mutation and prothrombin gene mutation, which could contribute to their increased risk of VTE [5,7].

The clinical presentation of PE can be variable and nonspecific, ranging from mild dyspnea to hemodynamic collapse [8]. Common symptoms include sudden-onset chest pain, shortness of breath, tachypnea, tachycardia, and hypoxemia [9]. Less common symptoms may include syncope, hemoptysis, and signs of DVT such as unilateral leg swelling [8,9]. The nonspecific nature of these symptoms can make PE difficult to diagnose, particularly in young patients who may not have obvious risk factors. Diagnosis relies on a combination of clinical assessment, laboratory testing (e.g., D-dimer), and imaging studies such as computed tomography pulmonary angiography (CTPA) or ventilation-perfusion scintigraphy [10]. Prompt recognition and treatment with anticoagulation are essential to prevent morbidity and mortality associated with PE [11].

Here, we present a case of acute PE in a young African American patient with no identifiable risk factors, highlighting the importance of maintaining a high index of suspicion for this condition even in low-risk populations. This case underscores the need for further research into the epidemiology, risk factors, and optimal management strategies for PE in young African Americans. A better understanding of the factors contributing to the increased risk of VTE in this population could help guide prevention and treatment efforts. Additionally, increased awareness among healthcare providers about the potential for PE in young, apparently healthy individuals may lead to earlier diagnosis and improved outcomes.

## Case presentation

A 33-year-old African American male with a medical history of asthma presented to the emergency department with penile pain, penile swelling, hematuria, left flank pain radiating to the left groin, and a penile rash persisting for one week. He was admitted for balanoposthitis with acute urinary retention and started on prophylactic heparin due to hospitalization. Notably, the patient had no significant comorbidities or risk factors for thromboembolism.

Despite being on prophylactic heparin, the patient's clinical course was complicated by two cardiac arrests secondary to pulmonary embolism (PE) on the fifth day of hospitalization. He was resuscitated, intubated, and transferred to the ICU, where he suffered a second cardiac arrest. Echocardiography revealed right ventricular dilation and McConnell's sign, indicative of massive PE. Fibrinolytic therapy with alteplase (100 mg over 2 hours) and a heparin infusion were initiated. However, his course was further complicated by anuric acute kidney injury requiring hemodialysis on Day 7 of hospitalization, as well as shock liver and gastrointestinal bleeding.

**Figure 1:**
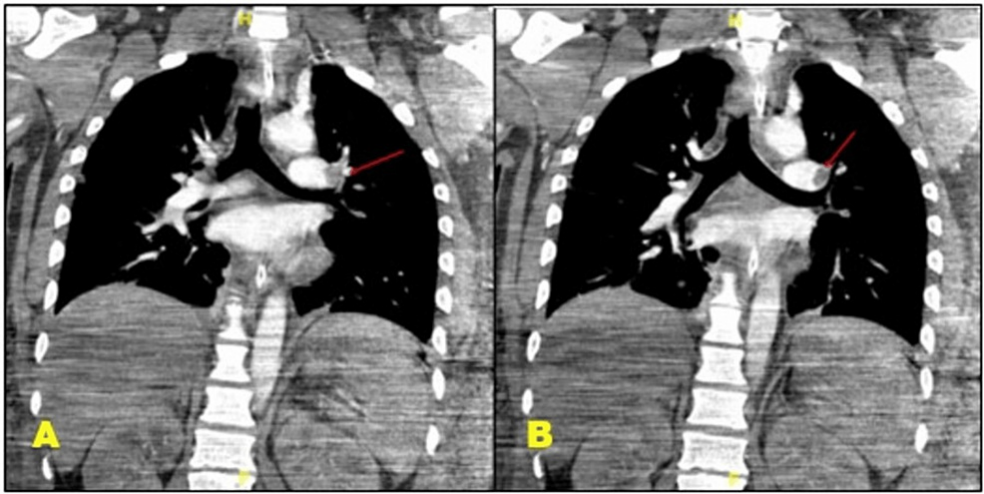
CT pulmonary angiography (coronal view) This coronal section of a CT angiogram highlights a pulmonary embolism with arrows pointing to the clot located in the pulmonary artery.

**Figure 2:**
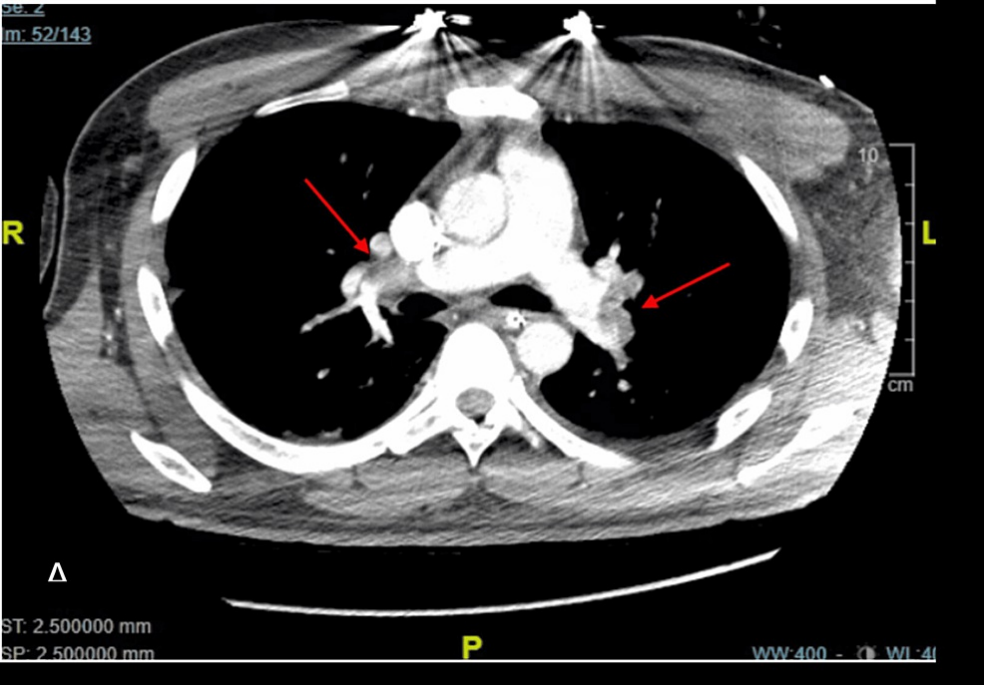
CT pulmonary angiography (axial view) This axial section of a CT angiogram clearly shows the presence of a pulmonary embolism, with arrows indicating the clot obstructing the pulmonary artery bilaterally.

Prior to imaging studies, laboratory tests were performed which revealed a significantly elevated D-dimer level of 170,000 ng/mL (normal range < 500 ng/mL). This finding, in conjunction with the patient's sudden clinical deterioration and echocardiographic evidence of right ventricular strain, raised a high suspicion for pulmonary embolism. Imaging studies were performed to assess the extent of thromboembolism. Venous Doppler showed only a small nonocclusive mural thrombus in the right common femoral vein, with no occlusive DVT. However, CT pulmonary angiography revealed bilateral pulmonary emboli with a large thrombus burden as shown in Figures 1-2. CT head demonstrated anoxic encephalopathy changes, likely secondary to the cardiac arrests.

The patient's social and family history were noncontributory. He denied tobacco and recreational drug use and reported only occasional alcohol consumption. His occupation as a cleaner was not associated with any apparent risk factors for thromboembolism.

After a prolonged hospital stay with supportive care and management of complications, the patient eventually recovered and was safely discharged. 

## Discussion

PE, a serious medical emergency, often arises from pulmonary artery obstruction by emboli, commonly originating from DVT [10]. While PE commonly manifests in conjunction with identifiable risk factors such as surgery, trauma, or underlying comorbidities, encountering unprovoked PE in young patients without apparent predisposing conditions is rare and perplexing. This challenges conventional understanding, emphasizing the need for hypervigilance and comprehensive investigation to elucidate underlying mechanisms.

Understanding the multifaceted nature of VTE, which includes PE, DVT, and superficial thrombophlebitis, necessitates consideration of various risk factors. Thrombosis typically results from the interaction of Virchow's triad factors, encompassing hypercoagulability, endothelial injury or trauma, and stasis [12]. While acquired risk factors such as recent surgery, trauma, immobility, pregnancy, oral contraceptives, smoking, obesity, atherosclerosis, hypertension, and hospital-acquired infections are common, malignancies, particularly hematological, lung, pancreatic, and brain cancers, pose a significant risk for PE. The interconnection between DVT and PE further underscores the complexity of these conditions.

This case illustrates the diagnostic challenges of PE in young patients without clear risk factors, especially when it occurs despite thromboprophylaxis and with a negative hypercoagulable evaluation. The atypical presentation with genitourinary symptoms and lack of conventional predisposing factors contributed to the diagnostic difficulty [4]. Exploring potential contributing factors necessitates a holistic evaluation encompassing genetic, environmental, and lifestyle considerations, along with an examination of rare causes like May-Thurner syndrome [13,14]. Genetic predispositions, particularly inherited thrombophilia such as antithrombin III deficiency, protein C and S deficiencies, and factor V Leiden mutation, have been implicated in cases of unexplained VTE. Hence, an in-depth analysis of coagulation profiles and molecular markers associated with thrombotic events is warranted [15]. Integrating these multifaceted considerations into diagnostic protocols can enhance the accuracy and efficacy of identifying and managing PE in young patients.

The occurrence of PE in a young patient without apparent predisposing conditions prompts critical reflections on preventive measures that hospitals could employ to avert subsequent cardiac arrest. Beyond individual risk factors, systemic strategies and interventions are vital for enhancing early detection, intervention, and overall prevention of such life-threatening events. Individualized thromboprophylaxis is increasingly recognized as essential in managing VTE, particularly when conventional risk factors may not fully explain thrombotic events. Acknowledging that each patient presents a unique profile of risk factors, comorbidities, and clinical circumstances, tailored thromboprophylaxis maximizes efficacy while minimizing adverse events [16]. Traditional risk assessment tools such as the Wells score may not fully capture nuanced risk factors. For instance, prior to the acute cardiac events, this patient had a Wells score of 1.5 points, categorizing him into a low-risk group with a 1.3% chance of PE in an emergency department population [17]. Factors like obesity and African American race may also influence the efficacy of standard prophylactic regimens [18,19]. Integrating clinical prediction models, biomarkers, and imaging techniques can further refine risk stratification methodologies [17].

The case reported by Walker et al. shares key similarities with our case, as both involve young patients presenting with PE and right ventricular strain, highlighting the importance of considering PE even in younger populations without apparent risk factors [16]. The study by Zakai et al. provides important context for understanding the increased risk of VTE in African Americans, particularly in younger age groups, which is relevant to our case involving a young African American male [5]. These comparisons help situate our case within the larger body of knowledge on PE in young, apparently low-risk patients and emphasize the need for heightened vigilance and thorough diagnostic evaluation in such scenarios.

Preventing PE in young patients without apparent predisposing conditions necessitates systemic strategies beyond individual risk factors. Customized and individualized thromboprophylaxis, incorporating clinical prediction models, biomarkers, and imaging, optimizes risk assessment. Complementary non-pharmacological measures such as mechanical compression devices and early mobilization augment pharmacological interventions. Advanced imaging techniques, interdisciplinary collaboration, patient education, continuous monitoring, and refined imaging protocols are paramount for enhancing PE management and prevention.

Maintaining a high index of suspicion for PE, even in the absence of classic risk factors and with negative hypercoagulable testing, remains crucial. Atypical symptoms such as syncope, infections, persistent tachycardia, or unexplained hypoxia warrant prompt evaluation, underscoring the importance of a multidisciplinary approach involving pulmonology, hematology, and radiology for timely diagnosis and management.

## Conclusions

This case underscores the diagnostic challenges of PE in young patients without overt risk factors, especially when it occurs despite thromboprophylaxis and with negative hypercoagulable testing. Atypical presentations and lack of classic predisposing factors can delay diagnosis. Maintaining a high index of suspicion, utilizing a comprehensive diagnostic approach, and employing individualized thromboprophylaxis strategies are crucial for timely recognition and management of PE in such challenging scenarios. Further research is needed to refine risk assessment tools and prophylaxis protocols for young, low-risk patients with negative hypercoagulable evaluations.
